# TETRALEC, Artificial Tetrameric Lectins: A Tool to Screen Ligand and Pathogen Interactions

**DOI:** 10.3390/ijms21155290

**Published:** 2020-07-25

**Authors:** Silvia Achilli, João T. Monteiro, Sonia Serna, Sabine Mayer-Lambertz, Michel Thépaut, Aline Le Roy, Christine Ebel, Niels-Christian Reichardt, Bernd Lepenies, Franck Fieschi, Corinne Vivès

**Affiliations:** 1 Institut de Biologie Structurale, CEA, CNRS, University of Grenoble Alpes, F-38000 Grenoble, France; silviaachilli20@gmail.com (S.A.); michel.thepaut@ibs.fr (M.T.); aline.le-roy@ibs.fr (A.L.R.); christine.ebel@ibs.fr (C.E.); franck.fieschi@ibs.fr (F.F.); 2 Immunology Unit & Research Center for Emerging Infections and Zoonoses (RIZ), University of Veterinary Medicine Hannover, 30559 Hannover, Germany; terenomonteiro.joao@mh-hannover.de (J.T.M.); sabine-mayer1@gmx.de (S.M.-L.); Bernd.Lepenies@tiho-hannover.de (B.L.); 3 Glycotechnology Laboratory, Center for Cooperative Research in Biomaterials (CIC biomaGUNE), Basque Research and Technology Alliance (BRTA), CIBER-BBN, Paseo Miramón 182, 20014 San Sebastian, Spain; sserna@cicbiomagune.es (S.S.); nreichardt@cicbiomagune.es (N.-C.R.)

**Keywords:** C-type lectin, DC-SIGNR, glycan array, multivalency, pathogen recognition

## Abstract

C-type lectin receptor (CLR)/carbohydrate recognition occurs through low affinity interactions. Nature compensates that weakness by multivalent display of the lectin carbohydrate recognition domain (CRD) at the cell surface. Mimicking these low affinity interactions in vitro is essential to better understand CLR/glycan interactions. Here, we present a strategy to create a generic construct with a tetrameric presentation of the CRD for any CLR, termed TETRALEC. We applied our strategy to a naturally occurring tetrameric CRD, DC-SIGNR, and compared the TETRALEC ligand binding capacity by synthetic N- and O-glycans microarray using three different DC-SIGNR constructs i) its natural tetrameric counterpart, ii) the monomeric CRD and iii) a dimeric Fc-CRD fusion. DC-SIGNR TETRALEC construct showed a similar binding profile to that of its natural tetrameric counterpart. However, differences observed in recognition of low affinity ligands underlined the importance of the CRD spatial arrangement. Moreover, we further extended the applications of DC-SIGNR TETRALEC to evaluate CLR/pathogens interactions. This construct was able to recognize heat-killed *Candida albicans* by flow cytometry and confocal microscopy, a so far unreported specificity of DC-SIGNR. In summary, the newly developed DC-SIGNR TETRALEC tool proved to be useful to unravel novel CLR/glycan interactions, an approach which could be applied to other CLRs.

## 1. Introduction

Glycans play critical roles in many biological processes ranging from the maintenance of cell or tissue structure, molecular signal transduction, to cell recognition. The mechanisms by which they perform these diverse functions involve the interaction of the glycan with other endogenous or exogenous molecules. For instance, many cell-cell interactions are carbohydrate driven [1]. Detection of pathogens such as viruses, fungi and bacteria is mediated by the recognition of glycans expressed on the microorganism surface. Indeed, the human immune system possesses pattern recognition receptors (PRRs), expressed on dendritic cells, which are able to recognize molecular motifs and activate immunity [2]. Amongst those receptors, C-type lectin receptors (CLRs) are carbohydrate-binding proteins that are specifically involved in the recognition and the uptake of altered-self and non-self glycans through their Ca^2+^ dependent carbohydrate recognition domain (CRD) [3]. The crucial roles of CLRs in the balance of immunity make CLR-glycan interactions attractive for pharmaceutical interventions [4].

Human CLRs are generally characterized by a low affinity for their glycan partners. The single interaction between the protein and the isolated monosaccharide or small oligosaccharide is usually of low affinity, in the millimolar range [5]. This apparent drawback results from the necessity to recognize a set of different ligands which include a common recognition motif. The globular structure of the CRD, in fact, does not contain any cavity, therefore the recognition of carbohydrates occurs through a largely open binding site, centered on the Ca^2+^, limiting in many cases the level of selectivity achieved.

Nature compensates low affinity with the concerted force of multiple binding events. The accumulation of weak affinity forces leads to an apparent strong interaction, an effect called avidity [6]. Multivalent binding plays a crucial role in the cell-surface recognition and could be reached by different means. On the one hand, oligomerization can be achieved on the CLR side by either clustering of single CLR into membrane micro domains, multiple CRDs along a single polypeptide chain [7] or through oligomerization of CRD-containing receptors [8]. In some cases an oligomerization domain, termed neck, serves as a stalk to bundle several CRDs and project them from the cell membrane [9].

On the other hand, multivalent ligands also participate in the high-avidity binding and contribute to the “glycan cluster effect” that occurs when multivalency is achieved for both protein and glycan presentation. The interaction of DC-SIGN, a CLR expressed on dendritic cells (DCs), with its ligands remarkably illustrates this multivalency enhancement. Firstly, DC-SIGN is expressed on cell surfaces in a tetrameric form and is further clustered into microdomains in lipid rafts, leading to a dense presentation of the binding sites at the cell surface [10]. Secondly, surface glycans of the pathogens recognized by DC-SIGN, as for instance the high mannose type glycans of HIV envelope glycoprotein gp120, are also presented with an unusual density [11].

In vitro assays performed to study CLR/glycan interactions have to be technically adapted to mimic this multivalency, otherwise they would fail to reveal low affinity monovalent binding events. At the ligand level, one way to increase valency is to present artificial multivalent ligands. Those would include both low valency compounds, such as short polymers, glycoclusters or peptide conjugates and high valency compounds, such as dendrimers, liposomes or nanoparticles [12]. Another platform for the presentation of glycans with adjustable density for biological sample screening are glycan arrays, which consist of libraries of immobilized glycan structures spatially organized on a Supporting Material. On the protein side, lectin arrays [13,14], that consist in spatially addressable collections of immobilized lectin probes with known binding specificity, and surface plasmon resonance based assays, that employ lectin functionalized sensorchips [15], provide a multivalent lectin presentation for the study of interactions between glycoconjugates and lectins immobilized on a surface. In solution studies of protein/glycan molecular interactions can provide complementary information to surface based assays. However, when the natural protein only displays monomeric CRD, the artifices mentioned above to build up protein multivalency have to be replaced by the creation of soluble artificial multimeric lectins. One strategy commonly used is the creation of Fc-constructs. This approach based on the fusion of a CRD domain with an immunoglobin Fc domain enables dimerization [16] of the glycan binding site. Moreover, artificial tetramers have been created by the biotinylation of the CRD followed by conjugation to tetrameric streptavidin [17]. Here, we propose an alternative strategy to create a tetrameric complex of any lectin. The strategy is based on a site-specific biotinylation at the N-terminus of lectin CRD exploiting the “sortagging” labeling approach using the enzyme sortase A (SrtA) [18]. The resulting biotinylated CRD is then complexed with tetrameric NeutrAvidin to obtain the final molecule exposing four glycan binding sites named hereafter TETRALEC.

**Figure 1:**
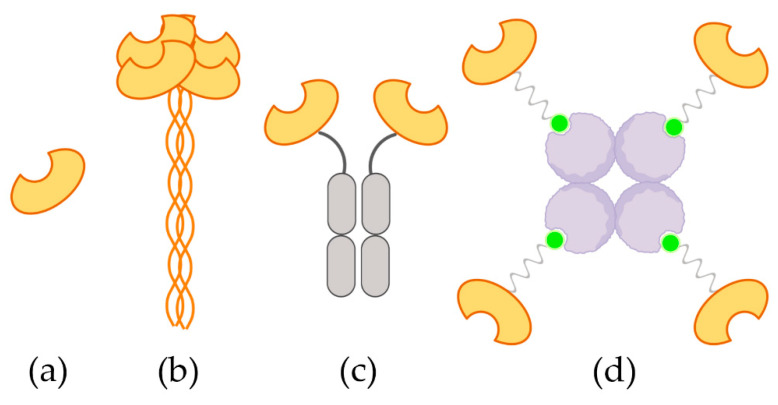
Schematic representation of the four constructs considered in this work. (**a**) Monomeric carbohydrate recognition domain (CRD); (**b**) natural extracellular domains (ECD) tetramer; (**c**) artificial dimeric Fc-CRD and (**d**) artificial tetrameric TETRALEC.

To validate the approach we decided to compare the functionality of a naturally existing tetrameric lectin, termed DC-SIGNR [19], with its artificial tetrameric counterpart. Lymph node-specific intercellular adhesion molecule-3-grabbing integrin or DC-SIGN related (L-SIGN or DC-SIGNR) is expressed in endothelial cells of lymph nodes, liver and placenta and shares 77% of amino acid homology with DC-SIGN [20]. Both are calcium-dependent mannose-specific CLRs that act as cell adhesion and endocytic receptors involved in the recognition of a broad range of viral and bacterial pathogens [21]. DC-SIGNR and DC-SIGN natural extracellular domains (ECD) present a tetrameric conformation exposing four CRDs [22]. Therefore, a direct comparison in terms of ligand interaction experiments of the ECD with the TETRALEC construct could be performed. Two other DC-SIGNR constructs were analyzed in parallel: the monomeric DC-SIGNR CRD in order to evaluate the benefit of an oligomeric presentation and a more routinely used artificial construct, in other words, a dimer formed by an Fc fusion with DC-SIGNR CRD (Figure 1). We have first characterized the oligomeric status of all the multimeric constructs. For all constructs we then compared the glycan binding profiles on a microarray of synthetic N- and O- glycans to evaluate the impact of CRD presentation and geometry on the avidity-based recognition process. Besides, we also studied the interaction between DC-SIGNR and *Candida albicans* by flow cytometry to show that our strategy could be used to screen new host/pathogen interactions.

## 2. Results

### 2.1. Design and Synthesis of the TETRALEC

**Figure 2:**
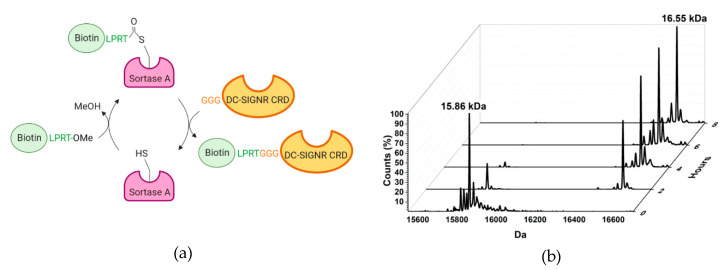
N-terminal biotin sortagging of DC-SIGNR-CRD. (**a**) Schematic representation of the reaction. (**b**) DC-SIGNR-CRD labeling followed by ESI-MS. The peak at 15,862 kDa corresponds to un-labeled CRD and the peak at 16,556 kDa corresponds to biotinylated CRD (15,862 + 0.694 kDa). After 6 h of reaction, the biotinylation reaction is completed.

In the Gram-positive *Staphylococcus aureus*, SrtA catalyzes the anchorage of target proteins, including virulence factors, to the cell wall. This enzyme cleaves the LPXTG motif present in the proteins and links them to the amino terminal group of five glycines of the peptidoglycan [23]. Here, SrtA was used to enzymatically couple a biotinylated peptide to the N-Terminus of the protein CRD previously extended with a poly-Gly chain (GGG) (Figure 2a). The peptidic motif LPRT-OMe recognized by the sortase A (SrtA) was appended to the biotin. Methylation of the terminal threonine residue drastically reduces the reversibility of the reaction [23].

The biotin sortagging was followed by electrospray ionization mass spectrometry (ESI-MS). An intermediate thio-ester sortase-biotin was initially formed, followed by the transpeptidation of the biotinylated peptide onto the DC-SIGNR GGG-CRD N-terminus. As shown in Figure 2b the reaction could be considered completed after 6 h of reaction.

Biotin sortagged DC-SIGNR-CRD was then purified and complexed via its biotin moiety to a tetramer of NeutrAvidin previously labeled with Cy3 fluorophore.

### 2.2. TETRALEC Structural Characterization

Size exclusion chromatography coupled to light scattering (SEC-LS) analysis was performed to assess the tetrameric presentation of DC-SIGNR-CRD by one molecule of tetrameric NeutrAvidin on DC-SIGNR TETRALEC, and verify DC-SIGNR ECD and DC-SIGNR Fc-CRD association states. SEC-LS combines the separation of macromolecules by size exclusion chromatography and their characterization in terms of molar mass and composition thanks to different detectors, static and dynamic light scattering, refractive index and absorbance.

**Figure 3:**
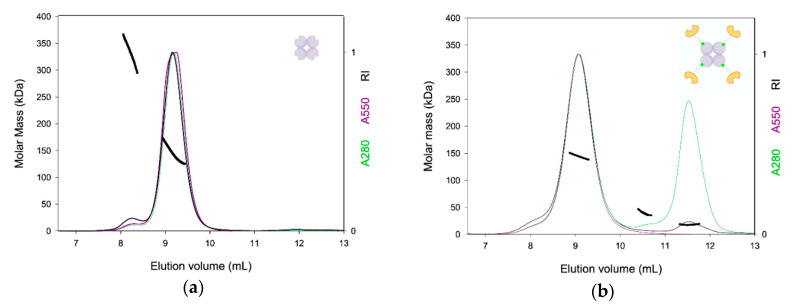
SEC-LS analysis of NeutrAvidin and TETRALEC. NeutrAvidin (**a**) and TETRALEC (**b**) normalized chromatograms at 280 nm (green), at 550 nm (purple), refractive index (RI) changes (black), and molar mass (thick lines) along the elution profiles. Molar masses were determined by combining refractive index and light scattering.

For analyzing TETRALEC, in addition to the refractive index detection, acquisitions at two wavelengths were performed: 280 nm, which probes both Cy3- NeutrAvidin and DC-SIGNR CRD, and 550 nm, which only probes Cy3- NeutrAvidin. Probing for Cy3- NeutrAvidin alone, the protein eluted as a main peak at 9.2 mL with a small shoulder at 8.2 mL containing larger species (Figure 3a). The extracted molar mass along the peak decreased slightly with the elution volume revealing some heterogeneity, and reached, after 9.4 mL, a value of around 120 kDa corresponding to a dimer of the natural tetramer of NeutrAvidin (MWtheo = 2 × 58 kDa).

The TETRALEC complex eluted with a main peak at 9.1 mL, and a shoulder at 8 mL (Figure 3b). Two additional contributions, which do not absorb at 550 nm, were detected at 10.5 mL and 11.5 mL, with molecular masses of 39 kDa and 18 kDa, corresponding most probably to free dimer and monomer DC-SIGNR CRD (MWtheo = 16.5 kDa), respectively. They are however in minor amounts as indicated by the refractive index signal (black line in Figure 3b). The main peak at 9.1 mL shows a slightly decreasing molar mass with the elution volume, which could be due to a contamination by the larger species from the shoulder. The mean molar mass is about 140 kDa, close to the MWtheo = 124 kDa calculated for the 4:4 Cy3- NeutrAvidin:DC-SIGNR CRD complex. The analysis in terms of stoichiometry could not be done with certainty. Considering the theoretical extinction coefficient at 280 nm for NeutrAvidin-Cy3, the analysis supports a 4:4 complex. Unfortunately, the analysis at 550 nm provides improbable stoichiometries ranging going from 7:2 to 5:3 of Cy3- NeutrAvidin:DC-SIGNR CRD. The discrepancy between the analyses should be related to uncertainty in one or the other extinction coefficients. We consider that the presence of free Cy3- NeutrAvidin is unlikely because of the evidence, at 11.5 mL, of free biotinylated DC-SIGNR CRD in excess. The SEC-LS experiment presented here cannot, by itself, give a definitive conclusion but strongly supports the formation of the 4:4 TETRALEC stoichiometry.

**Figure 4:**
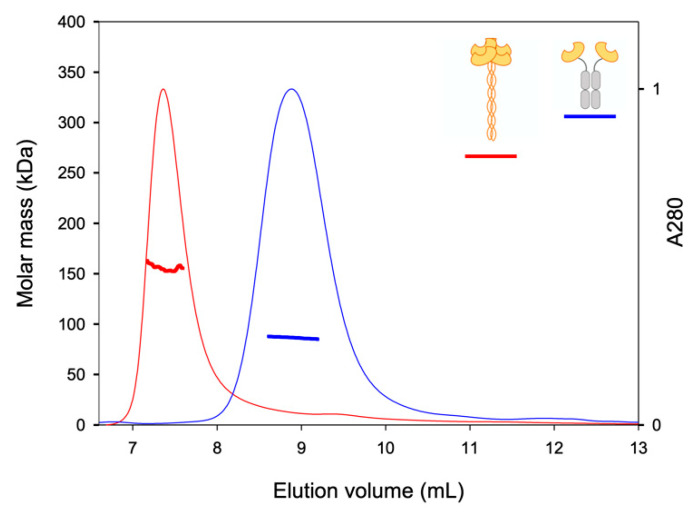
SEC-LS analysis of ECD construct (red) and Fc-CRD construct (blue). Normalized chromatograms at 280 nm (thin lines), and molar mass (thick lines) along the elution profiles. Molar masses were determined by combining refractive index and light scattering.

Finally, we controlled the association states of the two other constructs (Figure 4). The artificial construct DC-SIGNR Fc-CRD (blue lines) gave a main contribution at 8.9 mL, with a *M*_W_ of 86.5 kDa and a RH of 9 nm. This contribution corresponds to a dimer of DC-SIGNR-Fc-CRD (MWtheo = 79.4 kDa). The natural tetrameric DC-SIGNR ECD (red lines) gave a main contribution at 7.4 mL, with a *M*_W_ of 155.6 kDa, close to the theoretical value (MWtheo = 148.8 kDa) and a RH of 7.7 nm. These results confirmed the dimeric nature of Fc-CRD and the tetrameric nature of DC-SIGNR ECD.

### 2.3. Validation on Glycan Array

**Figure 5:**
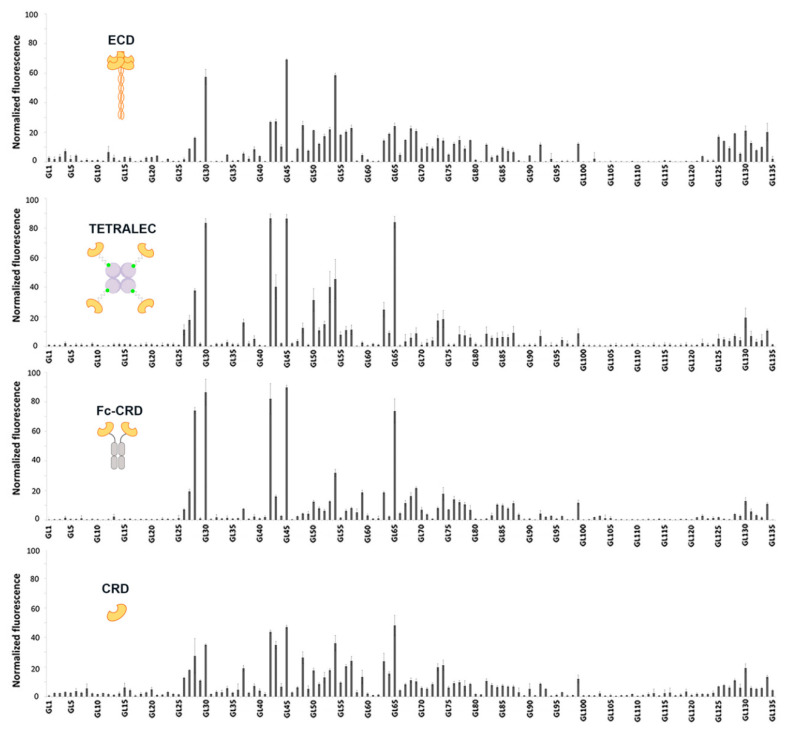
Glycan binding profiles of DC-SIGNR ECD, TETRALEC, Fc-CRD and CRD. Histograms representing the normalized fluorescence values. X-axis has been labeled with glycan numbers in ascending order with only one out of five glycan names mentioned.

Once the oligomeric status was determined, the glycan binding profile of the constructs was assessed on glycan microarrays. The objectives were i) to examine whether the artificial constructs were functional and ii) to appraise the effect of the oligomeric status and spatial arrangement of DC-SIGNR CRDs in glycan recognition. DC-SIGNR natural ligands are N-linked high-mannose oligosaccharides, presented on several pathogens. In comparison to DC-SIGN, fucosylated oligosaccharides, such as Lewis A and Lewis X blood group epitopes found on some pathogens are not recognized [24]. Thus, a synthetic glycan microarray (See SM, Figure S1) with 135 carbohydrates containing N-glycan and O-glycan structures (See SM, Table S1) was used to compare the different DC-SIGNR constructs [25,26]. DC-SIGNR CRD, ECD and TETRALEC were all labeled with Cy3 fluorophore while DC-SIGNR Fc-CRD binding was detected with Cy3 labeled secondary anti-human Fc antibody. The concentration of all constructs was adjusted to the same concentration of active CRD sites in each experiment. For this reason, while a concentration of 1 µM of the tetrameric DC-SIGNR ECD and TETRALEC was fixed for the incubation, 4 µM and 2 µM were used for the monomeric DC-SIGNR-CRD and the dimeric DC-SIGNR Fc-CRD, respectively. All constructs showed important recognition towards structures presented on glycan microarray (see structures in SM, Figure S1), highlighting their functionality (Figure 5). As equal amounts of DC-SIGNR CRD sites from different constructs were employed in microarray experiments, total intensity normalization was applied to the data to overcome the difference in labeling between the detection methods employed [27].

**Figure 6:**
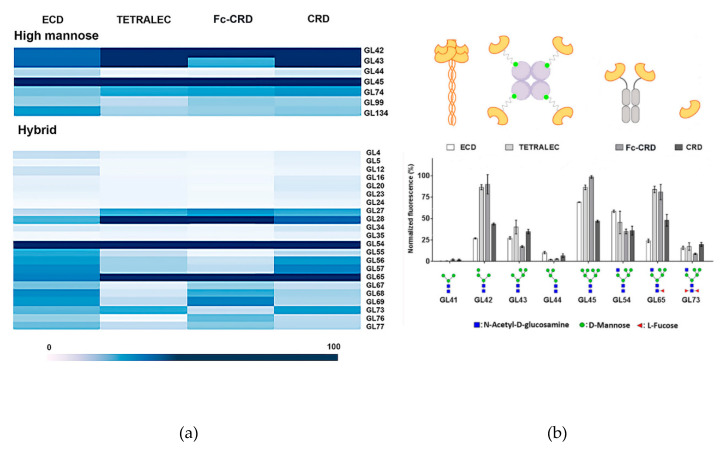
Glycan binding profiles of DC-SIGNR ECD, TETRALEC, Fc-CRD and CRD towards selected ligands. (**a**) High mannose and hybrid type N glycan structures were represented in a heat map classification. (**b**) Histograms representing the normalized fluorescence values.

As binding assays were performed only at a single protein concentration for each construct, affinity constants for individual interactions could not be determined. Nevertheless, the normalized fluorescence values indicate a ranking of affinities. All constructs exhibited a similar trend in glycan recognition, high mannose and hybrid N-glycans being the preferential bound structures (Figure 6a). It is important to highlight that certain differences in binding were also observed, underlining the importance of geometrical presentation and distance between CRDs for the recognition of epitopes (Figure 6) [28].

Natural tetrameric DC-SIGNR ECD showed recognition towards mannose containing N-glycan structures presented on the microarray, in line with previous reported specificity. The strongest binder was GL45, this high mannose N-glycan structure possesses an atypical non-natural branching pattern presenting in both antennae terminal Manα1-3(Manα1-6Man) trisaccharide residues, a well-known binding epitope for DC SIGNR [29]. The recognition towards high mannose structures is reduced with the number of mannoses residues (GL42, GL43, GL44 versus GL45, Figure 6b), as large high mannose structures are recognized by the extended primary binding site or via interaction with secondary binding sites at different positions of DC-SIGNR [29]. The interaction with paucimannose structures (Man1-3GlcNAc2) (GL41, Figure 6b) was negligible, due to the binding impediment with the primary binding site of DC-SIGNR caused by the presence of core β-linked GlcNAc attached to mannose residues [30]. The multivalent presentation of carbohydrates on the microarray surface enables efficient simultaneous binding with several closely immobilized carbohydrates, and indeed even monomeric DC-SIGNR CRD showed relevant binding strength, with a similar recognition profile as ECD. The artificial CRD tetramerization in the TETRALEC led to an increased specificity in ligand recognition compared to the natural ECD tetramer and CRD monomer. While the ECD construct binds to a broader range of ligands, narrower selection of glycans was strongly recognized by DC-SIGNR TETRALEC (Figure 5). The DC-SIGN Fc-CRD construct followed the same tendency of recognition observed for the other three constructs and share the increased specific recognition for some ligands with the TETRALEC. Avidity results from the combination of both ligand rebinding and multiple attachment (chelation). All CRDs are densely packed and have the same orientation in the ECD [31,32]. Thus, this construct is probably more favorable to multiple attachment than the TETRALEC or Fc-CRD presentations where the CRDs are more distant from one another. Indeed, even if the best ligands are the same for all different constructs, the differences observed in the glycan binding profile of the weaker ligands may reflect their capacity to undergo rebinding or simultaneous multiple attachment between the CRDs.

Nevertheless, one crucial result is that all ligands recognized by DC-SIGNR TETRALEC and Fc-CRD are actual ligands of the natural constructs (ECD or CRD) indicating that the artificial oligomerization of CRDs is not affecting the binding selectivity of DC-SIGNR. For certain glycans however, the oligomerization of CRD in the artificial constructs (Fc-CRD and TETRALEC) increases binding probably by the selection of certain glycans over others by the newly multimeric architectures. For example, GL42 (Figure 6b), that displays a Manα1,2-Man disaccharide on the non- reducing end, is stronger bound by both Fc-CRD and TETRALEC compared to ECD.

Additionally, we observed how core fucose, which is not part of the recognition element, influences binding to DC-SIGNR. For example, the three hybrid type N-glycan structures with and without core fucosylation (GL54, GL65 and GL73, Figure 6b) were bound differently by the constructs. Glycan GL54 displaying a terminal Manα1-3(Manα1-6Man) displayed similar binding strengths to the four constructs, with ECD being the strongest binder. Core α-1,6 fucosylation in GL65 leads to the reduction in binding to the natural ECD but to an increase in binding to the artificial constructs Fc-CRD and TETRALEC. On the other hand, the presence of both core α-1,6 fucose and core α-1,3 fucose (GL73) reduces binding with all four constructs. The role of core fucose as a key modulator of N-glycan binding to lectins has been recognized previously and attributed to the conformational changes in the oligosaccharide conformation induced by this modification [33,34].

### 2.4. DC-SIGNR Recognizes Heat-Killed Candida albicans

**Figure 7:**
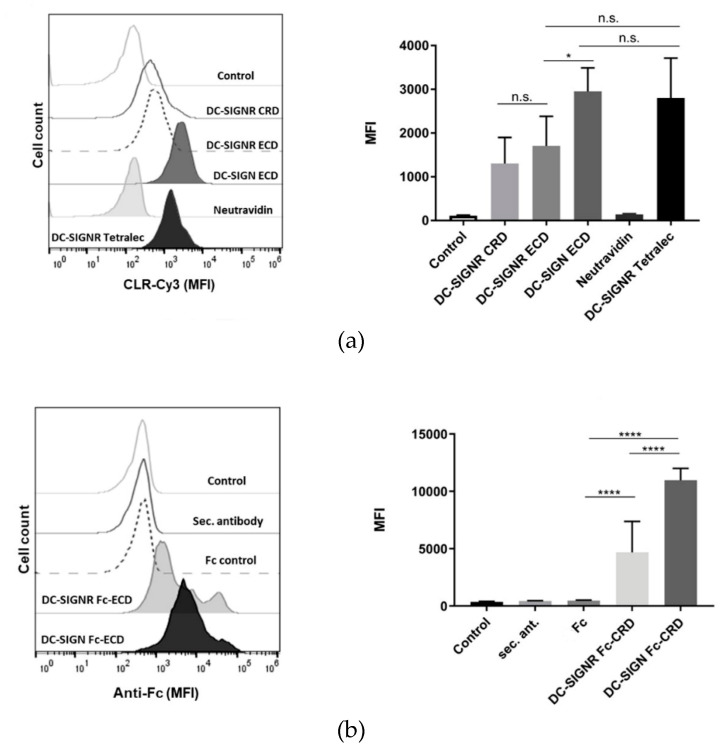
Heat-killed *Candida albicans* recognition by DC-SIGNR. (**a**, left) Representative experiment of the histograms obtained for heat-killed *C. albicans* (HKCA) binding by the different DC-SIGNR-Cy3 constructs. DC-SIGN ECD was used as a positive control. (**a**, right) Mean fluorescent intensity (MFI) of DC-SIGNR-Cy3 constructs binding to HKCA. Data depicted are the average of at least three independent experiments. (**b**, left) Representative experiment of Fc-CRD fusion proteins recognition of HKCA. Fc and the secondary antibody were used as negative controls, while DC-SIGN Fc-CRD is the positive control. (**b**, right) Average of the MFI values obtained. Data showed are the average of at least five independent experiments. Statistical analysis of the MFI results was performed using the unpaired Student’s *t* test, where p-values of <0.05 were considered to be significant (n.s. = not significant, * *p* ≤ 0.05, **** *p* ≤ 0.0001).

After assessing the capacity of the TETRALEC construct to recognize synthetic glycans, the possibility to use this tool to investigate CLR/pathogen interactions was explored. For this purpose, binding of the different CLR constructs to the pathogenic fungus *C. albicans*, a pathogen covered by N-glycans at its surface, was evaluated by flow cytometry. DC-SIGN was used as a positive control, based on its well-described role in the recognition of *C. albicans* and anti-fungal immune responses [35,36,37]. Binding to heat-killed *C. albicans* (HKCA) was observed for all DC-SIGNR-Cy3 constructs, in other words, DC-SIGNR CRD, ECD and TETRALEC (Figure 7a), albeit to a minor extent when comparing DC-SIGNR CRD and ECD to the positive control, DC-SIGN ECD.

NeutrAvidin did not impact binding to *C. albicans*, since no binding was observed when using the negative control Cy3-NeutrAvidin. HKCA was recognized by all DC-SIGNR-Cy3 constructs independently of their oligomeric status. The binding profile to HKCA was also evaluated with CLR Fc-CRD fusion proteins (Figure 7b). The Fc fragment works as a primary antibody, hence enabling detection of binding events with an anti-Fc antibody. Oppositely to DC-SIGNR-Cy3 constructs that present direct labeling of the CLRs, the CLR Fc-CRD fusion proteins employ an indirect labeling strategy. Thus, the labeling intensity cannot be directly compared to evaluate the impact of the dimeric presentation against the monomeric or tetrameric presentations for HKCA binding. DC-SIGNR Fc-CRD also showed binding to HKCA, although to a lower degree than DC-SIGN Fc-CRD (Figure 7b). The negative controls (Fc and secondary antibody alone) were not bound to HKCA, when compared with the CLR Fc-CRD fusion proteins.

**Figure 8:**
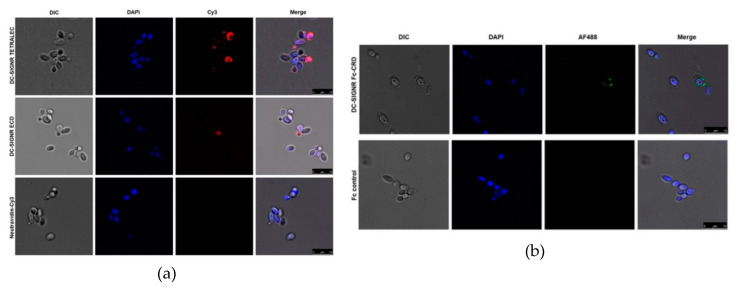
Binding of heat-killed *C. albicans* to different DC-SIGNR constructs using confocal microscopy. (**a**) Recognition of HKCA cell wall by DC-SIGNR TETRALEC (upper panels), DC-SIGNR ECD (middle panel) or NeutrAvidin-Cy3 (negative control, lower panels). (**b**) DC-SIGNR Fc-CRD binding to HKCA (upper panels) or Fc (negative control, lower panels). *C. albicans* cell wall was visualized by differential interference contrast (DIC). *C. albicans* DNA was stained using DAPI (in blue), while CLR-Fc constructs were detected using the AF488-conjugated secondary antibody (in green) and Cy3 detection of the DC-SIGNR-Cy 3 constructs is shown in red. Three random pictures were taken per independent experiment (*n* = 3). Scale bar indicates 10 µm.

Since we detected strong binding of DC-SIGNR TETRALEC and DC-SIGNR Fc-CRD to HKCA in a flow cytometry-based assay, we applied confocal microscopy to further visualize and confirm the identified interaction (Figure 8). The results obtained showed that both DC-SIGNR constructs interact with the cell wall of HKCA. Nevertheless, this binding appears weaker than the well-described DC-SIGN binding to *C. albicans* [37]. DC-SIGNR Fc-CRD interaction showed a punctuated behavior of binding to the cell wall of HKCA (Figure 8b), suggesting that the glycan structures recognized are not homogenously present at the surface of HKCA cell wall. DC-SIGN Fc-CRD, used as a positive control in the flow-cytometry experiments, showed a homogenous recognition of the entire surface of the HKCA cell wall (See SM Figure S2). In line with our observation of the DC-SIGNR Fc-CRD construct binding to HKCA, DC-SIGNR TETRALEC also exhibited a non-homogenous binding to HKCA cell (Figure 8a). It is noteworthy that virtually no binding was observed for the negative control employed for the CLR Fc-CRD constructs, the Fc control, (Figure 8b) and for NeutrAvidin-Cy3, the negative control of the DC-SIGNR TETRALEC construct (Figure 8a). Interestingly, DC-SIGNR TETRALEC showed a stronger interaction with HKCA than DC-SIGNR ECD by confocal microscopy (Figure 8a), strengthening the TETRALEC construct as a relevant tool to evaluate glycan recognition via glycan array and CLR/pathogen interactions using different methodologies.

## 3. Discussion

The crucial roles played by CLRs in many biological processes including pathogen recognition mechanism or modulation of immune response place them as strategic targets for pharmacological intervention. Moreover, as several CLRs remain “orphan” with no ligand identified, the characterization of their carbohydrate binding specificities is required prior to any drug development. While multivalent ligand or receptor presentation can be easily achieved in surface-based assays, increasing the valency of the protein to perform in solution studies is less obvious. A common option is the creation of Fc fusion proteins that enables dimerization of the glycan binding site. Such constructs have successfully been used to screen for new CLR pathogen ligands and have for instance evidenced the following interactions: Mincle/*Pneumocystis carinii* [38], Mincle/*Streptococcus pneumoniae* [39], SIGNR3/*Lactobacillus acidophilus* [40]. However, this method only provides limited oligomerization enhancement and a higher level of multivalency may be required for some applications. One way to create artificial tetramers is the random labeling of the protein with a biotin tag followed by its conjugation to tetrameric streptavidin. This strategy, however, presents two major drawbacks: i) the degree of labeling is random and ii) any of the accessible lysines can be targeted which may affect the lectin/sugar interactions. An alternative strategy has been developed by the Drickamer group who formed a complex between biotinylated lectin CRDs produced in *Escherichia coli* and streptavidin. Biotinylation of the lectin CRD was achieved by the addition of a 13 AA sequence at the C-terminal that contains a single lysine in an appropriate context, which can be biotinylated by the coexpressed bacterial biotin ligase BirA [41]. Complexation with streptavidin enabled the creation of a molecule presenting 4 CRDs. This strategy was employed to produce tetrameric mouse DC-SIGN-related proteins for interaction studies [42]. It was also used to study the interaction between artificial peptide-MHC oligomers with cell surface TCRs [43].

Here, we present an alternative route for the biotinylation of DC-SIGNR CRD via sortagging with SrtA. This strategy only requires the introduction of a few (in theory even a single) glycine residues at the N-terminus of the protein of interest and the transfer under mild conditions of literally any substrate carrying a LPXTG motif [44]. We irreversibly tagged DC-SIGNR CRD with a biotin carrying a C-terminal methyl ester (Biotin-LPXT-OMe). The reaction produces methanol instead of a short peptide with a N-terminal glycine preventing the competing back-reaction with the side product [18]. Another advantage of sortagging is that it requires minor sequence modification of the target protein compared to the BirA approach. Besides, this approach can also be employed to incorporate other functionalities to the N-terminus of CRDs and offers very versatile applications. The enzymatic activity of SrtA has been widely used to link fluorescent tags [45], glycosylphosphatidylinositol (GPI) mimics [46] or even PEG chains [46].

We also improved the homogeneity of the final TETRALEC complex by using NeutrAvidin for biotin chelation. NeutrAvidin is a deglycosylated tetrameric protein derived from avidin, its biochemical characteristics, including a near-neutral electrical point and no glycosylation, reduce non-specific binding compared to streptavidin [47].

Although similar artificial oligomeric constructs have been used to study Glycan/CLR interactions [38,39,40,41,42], to our knowledge they have not been thoroughly characterized before their use in binding studies. The objective of our approach was, besides a novel conception of artificial tetrameric lectins, to ensure that these synthetic constructs retain a capability to identify genuine ligands. Towards this end, we compared the DC-SIGNR TETRALEC glycan binding profile with the natural DC-SIGNR tetramer, the dimeric DC-SIGNR Fc-CRD and monovalent DC-SIGNR CRD binding domains. The oligomeric status of all lectin constructs was assessed by SEC-LS analysis that supports tetramer formation of the biotinylated NeutrAvidin construct.

Interestingly, the overall recognition glycan pattern was comparable for all constructs (Figure 5). The functionality of the TETRALEC construct is evidenced by the fact that its selectivity follows that of the CRD (Figure 6a) with no false positives. Besides, it provides a clear enhancement of binding measured as a gain in fluorescence intensity (2 to 3 times increase). Thus, the artificial tetramer represents a relevant tool to identify primary ligands and would present a valuable advantage over naturally non-oligomeric lectins for which in solution interaction studies are impaired by low affinity binding. The pattern recognition of the Fc-fusion construct is very comparable to that of the TETRALEC.

This systematic binding comparison of a large range of ligands to constructs offering different CRD presentations revealed that the overall selectivity is not affected, in other words, that all constructs share common ligands. However, binding to some glycans appears to be increased for some constructs as observed with GL42 or GL45 that are preferentially recognized by the two artificial multivalent constructs. This behavior suggests, on the one hand, that the number of CRDs matters as indeed the multiplicity of the CRDs within one molecule will obviously favor the interaction. On the other hand, our results suggest that the CRD specific spatial arrangement also impacts molecular recognition. In this sense, the binding variation observed between DC-SIGNR ECD that presents four CRDs pointing in the same direction and the TETRALEC construct with active sites pointing in four different directions could be explained. Besides, the dense and uniform glycan presentation of the glycan array may favor binding of the TETRALEC and therefore somehow differs from natural lectin-glycan interactions. In addition, the specificity of lectin-glycan interaction can result from the cross-linking of bivalent oligosaccharides with oligomeric lectins. These interactions, previously described, clearly depend upon the spatial geometry of the glycans and the CRDs [48,49]. As a matter of fact, binding differences between the various constructs are most probably due to variable contributions of the different active binding modes, in other words, rebinding and chelation that generate avidity.

We also wanted to assess whether the TETRALEC construct could be used in cellular assays and more precisely if it could help to identify new CLR/pathogen interactions. Like many pathogens, *C. albicans* encounter with host defense involves its detection and clearance by the innate immune system, where CLRs expressed on the surface of epithelia, endothelial and myeloid cells play a pivotal role. *C. albicans* has a unique and highly mannosylated cell wall, where N- and O- glycans account for more than 90% of the glycans present at the surface [50]. Within the context of a larger screening approach to identify CLRs capable of recognizing *C. albicans* glycans we focused on the possibility of DC-SIGNR to interact with this fungal pathogen. Using the three different Cy3 labeled constructs of DC-SIGNR (DC-SIGNR CRD, DC-SIGNR ECD, DC-SIGNR TETRALEC) we evaluated two main points: first, if DC-SIGNR is able to bind to HKCA and, secondly, the impact of multivalent CRD presentation on the pathogen recognition. All three constructs recognized HKCA. Moreover, the TETRALEC construct presented a similar binding to the one observed for the positive control used, the DC-SIGN ECD construct. On the contrary, NeutrAvidin-Cy3, the negative control, showed no interaction, indicating that this conjugation strategy represents a useful tool to identify novel CLR-pathogen interactions, with minimal unspecific binding. Interestingly, the different oligomerizations states of DC-SIGNR-Cy3 constructs did not significantly impact recognition of HKCA by flow cytometry. It is noteworthy that both DC-SIGN ECD and DC-SIGN-Fc-CRD constructs displayed a stronger interaction to HKCA by flow cytometry, when compared to the different DC-SIGNR constructs. These binding differences between DC-SIGN and DC-SIGNR to pathogens that possess high mannose oligosaccharides may be associated with the different capacities of the CLRs to accommodate the differential spatial conformations of sugar epitopes in their binding pockets due to the distinct properties of the neck domains [51]. Despite an overlap in mannosylated glycans recognition of DC-SIGNR with DC-SIGN [24], no binding of DC-SIGNR and fungi has yet been described to the best of our knowledge. Therefore, in addition to proposing a new strategy to create an artificial tetrameric lectin construct with a recognition specificity close to its natural tetrameric counterpart, this study has also highlighted the utility of this construct to probe CLR/pathogens interactions, resulting in the identification of *C. albicans* as a pathogen recognized by DC-SIGNR.

## 4. Materials and Methods

### 4.1. Cloning

Standard pUC57 plasmids containing optimized synthetic human genes encoding human DC-SIGNR ECD (amino acids 78 to 399) and CRD (amino acids 264 to 399) designed for the efficient production in *Escherichia coli* were manufactured by GeneCust (Boynes, France) PCR amplification using suitable primers and restriction enzyme digestion were used to sub-clone into the pET30-b (Merck, Damstadt, Germany) DC-SIGNR ECD between the NdeI and HindIII restriction sites and DC-SIGNR CRD between the XbaI and HindIII sites. The sequencing of each construction was done by Genewiz (Leipzig, Germany).

### 4.2. Protein Expression and Purification

DC-SIGNR ECD was expressed in *E. coli* BL21(DE3) in 1 L of LB medium supplemented with 50 μg·mL^−1^ kanamycin at 37 °C. Expression was induced by addition of 1 mM isopropyl 1-thio-D-galactopyranoside (IPTG) when the culture had reached an A_600 nm_ of 0.8 and maintained for 3 h. The protein was expressed in the bacterial cytoplasm as inclusion bodies. Cells were harvested by a 20 min centrifugation at 5000× *g* at 4 °C. The pellet was resuspended in 30 mL of a solution containing 150 mM NaCl, 25 mM Tris-HCl pH 8 and one anti-protease mixture tablet (Complete EDTA-free, Merck, Damstadt, Germany). Cells were disrupted by sonication and cell debris eliminated by centrifugation at 100,000× *g* for 45 min at 4 °C in a Beckman 45Ti rotor. The pellet was solubilized in 30 mL of 6 M guanidine-HCl containing 25 mM Tris-HCl pH 8, 150 mM NaCl and 0.01% (*v*/*v*) beta-mercaptoethanol. The mixture was centrifuged at 100,000× *g* for 45 min at 4 °C and the supernatant was diluted 5-fold with 25 mM Tris-HCl pH 8, 1.25 M NaCl and 25 mM CaCl_2_ by slow addition with stirring. The diluted mixture was dialyzed against 10 volumes of 25 mM Tris-HCl pH 8, 150 mM NaCl, 4 mM CaCl_2_ (buffer A) with 3 buffer changes. After dialysis, insoluble precipitate was removed by centrifugation at 100,000× *g* for 1 h at 4 °C. The supernatant containing DC-SIGNR ECD was loaded on Mannan agarose column (Merck, Damstadt, Germany) equilibrated with buffer A for purification by affinity chromatography. After loading, DC-SIGNR ECD was tightly bound to the column and eluted in the same buffer without CaCl_2_ but supplemented with 1 mM EDTA (buffer B). This step was followed by SEC (size exclusion chromatography) using a Superose 6 column (Cytivia, Velizy-Villacoubray, France) equilibrated with buffer A. Fractions were analyzed by SDS-PAGE (12%) and DC-SIGNR ECD containing fractions were pooled and concentrated by ultrafiltration (YM10 membrane from Merck, Damstadt, Germany).

DC-SIGNR CRD was expressed in *E. coli* BL21(DE3) in 1 L of LB medium supplemented with 50 μg·mL^−1^ kanamycin at 37 °C. Expression was induced by addition of 1 mM IPTG when the culture had reached an A_600 nm_ of 0.8 and maintained for 3 h. The protein was expressed in the cytoplasm as inclusion bodies. Cells were harvested by a 20 min centrifugation at 5000× *g* at 4 °C. The pellet was resuspended in 30 mL of a solution containing 150 mM NaCl, 25 mM Tris-HCl pH 8 and one anti-protease mixture tablet. Cells were disrupted by sonication and cell debris eliminated by centrifugation at 100,000× *g* for 45 min at 4 °C in a Beckman 45Ti rotor. The pellet was solubilized in 30 mL of 6 M guanidine-HCl containing 25 mM Tris-HCl pH 8, 150 mM NaCl and 0.01% beta-mercaptoethanol. The mixture was centrifuged at 100,000× *g* for 45 min at 4 °C and the supernatant was diluted 5-fold with 200 mM Tris-HCl pH 8, 1.25 M NaCl and 25 mM CaCl_2_ by slow addition under stirring. The diluted mixture was dialyzed against 10 volumes of buffer A with 3 buffer changes. After dialysis, insoluble precipitate was removed by centrifugation at 100,000× *g* for 1 h at 4 °C. The supernatant containing the His-tagged DC-SIGNR CRD was loaded onto a HisTrap column (Cytivia, Velizy-Villacoubray, France) at 4 °C. Unbound proteins were washed away with buffer A before DC-SIGNR CRD was eluted with buffer C (150 mM NaCl, 25 mM Tris-HCl, pH 8, 4 mM CaCl_2_, 0.5 M imidazole). Eluted fractions were analyzed by SDS-PAGE (15%) and the DC-SIGNR CRD containing fractions were pooled and concentrated by ultrafiltration (YM10 membrane from Amicon).

Each construct was checked by N-terminal amino acid sequencing and mass spectrometry.

### 4.3. Labelling

A total of 250 μL of 3.77 mg·mL^−1^ solution of DC-SIGNR ECD, 212.5 µL of 4.75 mg·mL^−1^ solution of DC-SIGNR CRD in 25 mM HEPES pH 7.25, 4 mM CaCl_2_ and 100 µL of 3.47 mg·mL^−1^ solution of NeutrAvidin in PBS pH 7.4 were prepared. Then, 1 µL, 4 µL and 2 µL, respectively, of 10 mg·mL^−1^ Cy3-NHS ester (Gene Copoeia, Rockville, MD, USA) were added to the solutions and the reactions were gently shaken at room temperature for 2 h and then at 4 °C for 4 h. Excess dye was removed by two dialyses (3.5k Z-lyser from ThermoFisher Scientific, Waltham, MA, USA) of 3 h against 25 mM Tris pH 8, 150 mM NaCl, 4 mM CaCl_2_. The amount of attached Cy3 was estimated spectrophotometrically based on the dye molar extinction coefficient (ε = 150,000 cm^−1^·M^−1^) and the protein extinction coefficients. The obtained degrees of labeling (DOL) were 0.4, 0.2 and 0.5 for DC-SIGNR ECD, DC-SIGNR CRD and NeutrAvidin, respectively.

### 4.4. TETRALEC Formation

His tag cleavage. His-GGG-DC-SIGNR CRD was cleaved using factor Xa (ThermoFisher Scientific, Waltham, MA, USA) following the protocol recommended by the manufacturer: 1 µg of factor Xa per 50 µg of His-GGG-DC-SIGNR CRD protein at 1 mg·mL^−1^. The reaction was performed overnight at room temperature under agitation and then injected into Toyopearl exclusion chromatography column previously equilibrated in 25 mM Tris pH 8, 150 mM NaCl, 4 mM CaCl_2_ buffer. A flow rate of 1 mL·min^−1^ was maintained during the purification. Eluted fractions were pooled and concentrated up to 1 mg.

Biotin sortagging and TETRALEC formation. A protocol already published was used for GGG DC-SIGNR CRD biotinylation [18]. The protein exposing three glycines at the N-terminus (1 molar equivalent) was mixed with the biotin-LPRT-OMe peptide (*M*_W_ = 725.9 Da, Covalab, Villeurbanne, France) (5 eq.) and His-tag sortase A (SrtA) (0.3 eq.) from *Staphylococcus aureus*, recombinantly produced in the laboratory, in 25 mM Tris pH 8, 150 mM NaCl, 4 mM CaCl_2_ buffer. The reaction was incubated at 37 °C for 6 h under agitation. The kinetic of the reaction was followed by ESI-MS: 10 µL of reaction was analyzed at 0 h, 2 h, 4 h, 6 h, 8 h and overnight. When the reaction was completed, the solution was loaded onto a 1 mL HisTrap column previously equilibrated in 25 mM Tris pH 8, 150 mM NaCl, 4 mM CaCl_2_ buffer. After column washing, the elution step was performed using 25 mM Tris pH 8, 150 mM NaCl, 4 mM CaCl_2_, 0.5 M imidazole buffer. A 1 mL·min^−1^ flow rate of buffer was maintained during the purification. The His tagged sortase was retained by the HisTrap column while the untagged biotin-CRD was eluted during the washing step and was pooled and dialyzed against 25 mM Tris pH 8, 150 mM NaCl, 4 mM CaCl_2_ to eliminate un-reacted biotinylated-peptide.

Finally, NeutrAvidin (*M*_W_ = 14.5 kDa, ThermoFisher Scientific, Waltham, MA, USA) sample previously labeled with Cy3-fluorophore (2.9 mg·mL^−1^, DOL = 0.5) was mixed to biotin-CRD with a molar ratio of 1:1 and the reaction was incubated overnight at 4 °C under agitation. The obtained CRD-TETRALEC was frozen in liquid nitrogen for storage at −80 °C.

### 4.5. SEC-LS Analysis

SEC-LS experiments were conducted on a HPLC (Schimadzu, Kyoto, Japan) consisting of a degasser DGU-20AD, an LC-20AD pump, an autosampler SIL20-ACHT, a communication interface CBM-20A, a UV-Vis detector SPD-M20A and a fraction collector FRC-10A, a column oven XL-Therm (WynSep, Sainte Foy d’Aigrefeuille, France) and a static light scattering detector miniDawn Treos, a dynamic light scattering detector DynaPro NANOSTAR, a refractive index detector Optilab rEX (Wyatt, Santa-Barbara, CA, USA). Samples of 50 µL were injected on a KW 802.5 column (Shodex, Tokyo, Japan) equilibrated at 4 °C with 25 mM Tris pH 7.5, 150 mM NaCl, 4 mM CaCl_2_, at a flow rate of 0.5 mL·min^−1^. The analysis was made with the software ASTRA, v5.4.3.20 (Wyatt, Santa-Barbara, California, USA). It uses static light scattering and refractive index measured along the elution to derive experimental molar masses, and in the protein conjugate module, additional absorbance measurement to discriminate the molar mass of each partner in a two-component system. The refractive index increments, and extinction coefficients at 280 nm, were calculated from amino acid composition using the program Sedfit (freely available in https://sedfitsedphat.nibib.nih.gov). We combined the refractive index detection and the 550 nm detection to determine an experimental extinction coefficient for Cy3-NeutrAvidin: 2890 cm^−1^·(g/L)^−1^.

### 4.6. Glycan Array Analysis

Glycan microarrays were prepared as previously described [26]. Cy3 labeled constructs were diluted in incubation buffer (25 mM Tris-HCl, 150 mM NaCl, 4 mM CaCl_2_, pH 7.5 containing 0.5% (*w*/*v*) bovine serum albumin (BSA) and 0.005% (*v*/*v*) Tween-20). Protein solutions (200 µL per array) were used to incubate individual wells on a glycan array slide at 4 °C for 18 h. Arrays were washed with incubation buffer without BSA, water and dried in a slide spinner. For the detection of Fc-CRD, a solution of anti-human IgG (Fc specific)-Cy3 antibody (1:1000 dilution in binding buffer was incubated in the dark for 1h. Microarrays were washed and dried as previously described. Fluorescence measurements were performed on a microarray scanner (Agilent G2565BA, Agilent Technologies, Santa Clara, CA, USA) at 10 µm resolution. Quantification of fluorescence was performed by ProScanArray Express software (Perkin Elmer, Waltham, MA, USA) employing an adaptive circle quantification method from 50 µm (minimum spot diameter) to 300 µm (maximum spot diameter). Average RFU values with local background subtraction of four spots and standard deviation of the mean were reported using Microsoft Excel and GraphPad Prism software.

### 4.7. Generation of Human DC-SIGNR-Fc Fusion Protein

The production of human DC-SIGNR and DC-SIGN fusion proteins were performed as previously described [52,53]. Briefly, a human cDNA library was used as template (GE Dharmacon, Lafayette, CO, USA) and specific primers to amplify the CRD of DC-SIGNR were generated (Eurofins Genomics, Ebersberg, Germany). The DC-SIGNR and DC-SIGN primers were as follows: DC-SIGNR forward, gaattcctatcaagaactgaccgatttg; DC-SIGN forward, gaattcgtccaaggtccccagctccat; DC-SIGNR reverse ccatggattcgtctctgaagcaggc; and DC-SIGN reverse, ccatggacgcaggaggggggtttggggt. PCR was used to amplify the cDNA, followed by ligation into a pFuse-hIgG1-Fc expression vector (InvivoGen, San Diego, CA, USA). The DC-SIGNR-Fc expression vector was used to transiently transfect CHO-S cells with MAX reagent (Invitrogen, Darmstadt, Germany). After 4 days of transfection, the supernatant was collected and the fusion proteins were purified with a HisTrap protein G HP column (GE Healthcare, Piscataway, NJ, USA). Protein purity was confirmed by SDS-PAGE with subsequent Coomassie Blue staining. Western blot using an anti-human IgG-horseradish peroxidase (HRP) antibody (Dianova, Hamburg, Germany) was also performed to detect the presence of the fusion protein.

### 4.8. Flow Cytometry-Based Binding to C. albicans

Heat-killed *C. albicans* (InvivoGen, San Diego, CA, USA) was stained for 15 min with 1 µM of DNA-staining dye Syto61 (ThermoFisher Scientific, Darmstadt, Germany) at 4 °C. The samples were subsequently washed two times with 1× PBS. Then, samples were incubated for 1 h either with 250 ng of the respective Fc-CRD fusion proteins in lectin-binding buffer (50 mM HEPES, 5 mM MgCl_2_, 5 mM CaCl_2_, pH 7.4) or with 1 µM of the DC-SIGNR CRD, DC-SIGNR ECD and DC-SIGNR TETRALEC constructs in their respective lectin-binding buffer (25 mM Tris, 150 mM NaCl, 4 mM CaCl_2_, pH 8.0). After washing once with the lectin-binding buffer, the pellet was suspended in a 1:200 PE-conjugated goat anti-human Fc antibody (Dianova, Hamburg, Germany) and incubated for 20 min at 4 °C, for detection of the bound Fc-CRD fusion proteins. Finally, cells were washed two times and flow-cytometric analysis was performed using an Attune NxT Flow Cytometer (ThermoFisher Scientific, Darmstadt, Germany). The gating strategy applied was a first gate in the *C. albicans* population, followed by a single cell population gating for doublet exclusion. In the single cell population gate, Syto61 positive cells were selected and further analyzed for CLR binding. The same gating strategy was performed for all experimental conditions within one experiment. Flow cytometry data were analyzed using the FlowJo version 10 software (FlowJo, Ashland, OR, USA).

### 4.9. Confocal Fluorescence Microscopy

The preparation of slides for binding visualization of heat-killed *C. albicans* (Invitrogen, San Diego, CA, USA) with the different DC-SIGNR constructs was performed as described before, with minor modifications [53]. Briefly, cover slides (ThermoFisher Scientific, Darmstadt, Germany) were cleaned with 70% ethanol and coated with poly-L-lysine solution (Sigma-Aldrich, St. Louis, MO, USA) for 30 min at 60 °C. Following that, 2 × 10^7^ CFU/mL of HKCA was incubated for 1 h at 4 °C with 0.5 µg of DC-SIGNR Fc-CRD, DC-SIGN Fc-CRD and Fc control in lectin binding buffer. For the DC-SIGNR-Cy3 constructs, 2 µM of the constructs were incubated in lectin-binding buffer with HKCA for 1 h at 4 °C. Samples were washed two times with lectin-binding buffer and the CLR-Fc constructs were further incubated with 1:200 goat anti-human Fc Alexa Fluor (AF) 488-conjugated antibody (Dianova, Hamburg, Germany) at 4 °C for 1 h. The samples were then washed one time and applied in the poly-L-lysine-coated cover slides followed by 1 h incubation at 37 °C. Upon coating of the samples, slides were washed one time with 1× PBS, followed by a fixation step using 1% PFA for 1 h at RT. Finally, the cover slides were mounted on microscopic slides (Roth, Karlsruhe, Germany) with proLong gold antifade mountant containing DAPI (ThermoFisher Scientific, Darmstadt, Germany), sealed and stored at room temperature overnight. The next day, slides were visualized using a TCS SP5 confocal inverted-base fluorescence microscope (Leica, Nussloch, Germany) equipped with a HCX PL APO 63 × 1.4 oil immersion objective. At least three independent experiments were performed, each with three randomly selected pictures. Appropriate negative controls were also used, namely, the secondary antibody control for the Fc constructs and the NeutrAvidin-Cy3, as a control for the DC-SIGNR TETRALEC construct.

## 5. Conclusions

The strategy proposed enables the irreversible functionalization of CLR CRD N-terminus with a biotin moiety and thereafter, by coupling to a NeutrAvidin molecule the generation of artificial tetrameric CLRs. As a proof of concept, DC-SIGNR was chosen by virtue of the possibility of a direct comparison with the natural tetrameric ECD. After confirmation of its oligomeric state, the ability of the synthetic TETRALEC construct to preserve binding towards genuine ligands was tested. Comparison with the other constructs proved the TETRALEC functionality and revealed ligand binding enhancement compared to monovalent CRD presentation. This would be a valuable advantage for naturally non-oligomeric CLRs. The study also underlined that not only oligomery but also the CRD spatial presentation affects ligand recognition. The TETRALEC was also considered in in vitro studies, where flow cytometry and confocal microscopy were used to analyze the interaction between DC-SIGNR and the pathogenic *Candida albicans*. The results revealed the ability of DC-SIGNR to recognize some surface ligands on *Candida albicans*, proving that the strategy could be exploited to screen new host/pathogen interactions.

## Supplementary Materials

Supplementary materials can be found at https://www.mdpi.com/1422-0067/21/15/5290/s1.

## Abbreviations

CLR	C-type Lectin Receptor
CRD	Carbohydrate Recognition Domain
DC-SIGN	Dendritic Cell-Specific Intercellular adhesion molecule-3-Grabbing Non-integrin
DC-SIGNR or L-SIGN	DC-SIGN related or Lymph node-specific intercellular adhesion molecule-3-grabbing integrin
DC	Dendritic Cell
DOL	Degree Of Labelling
ECD	Extracellular Domain
ESI-MS	Electrospray ionization mass spectrometry
GPI	GlycosylPhosphatidylInositol
HKCA	Heat-Killed *C. albicans*
HRP	Horseradish Peroxidase
IPTG	IsoproPyl 1-Thio-D-Galactopyranoside
LB	Lysogeny broth
PRR	Pattern Recognition Receptors
SEC	Size Exclusion Chromatography
SEC-LS	Size Exclusion Chromatography coupled to Light Scattering
SrtA	Sortase A
