# Supplementary material for: TETRALEC, Artificial Tetrameric Lectins: A Tool to Screen Ligand and Pathogen Interactions
Source: Int J Mol Sci. 2020 Jul 25;21(15):5290. doi: 10.3390/ijms21155290 (PMC7432041; doi:10.3390/ijms21155290)
Supplement: Supplementary file 1 [file ijms-21-05290-s001.pdf]

## Supplementary Materials

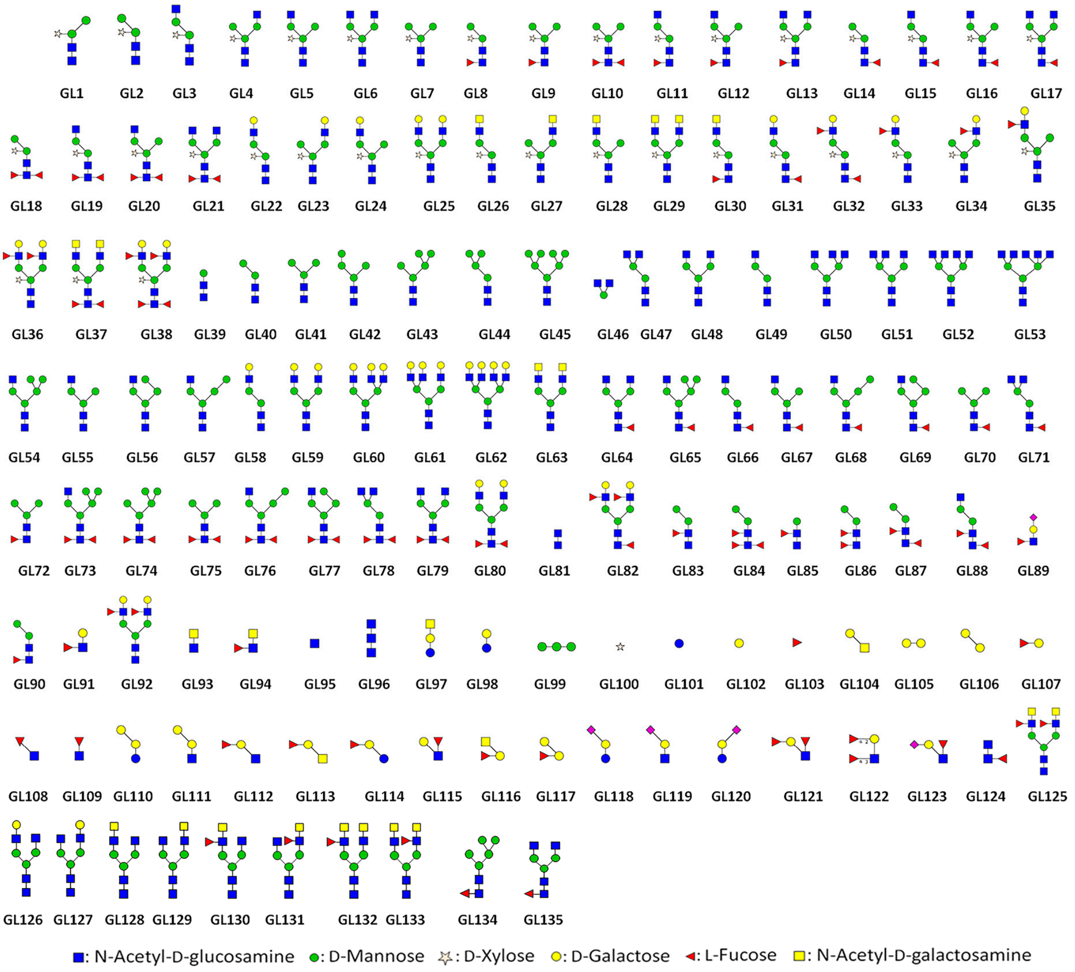


a

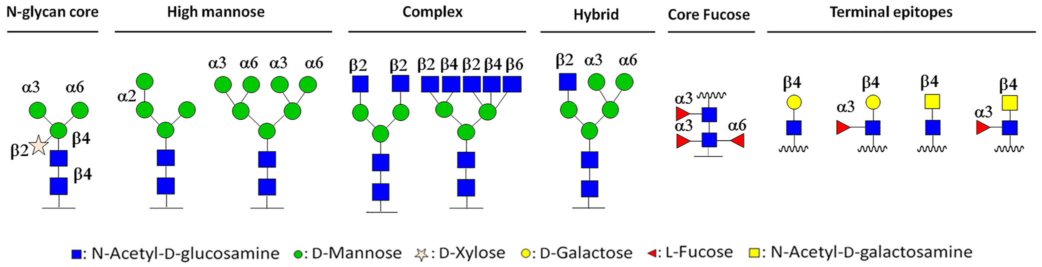


b

**Figure S1.** (**a**) Glycan structures included in microarrays. (**b**) Glycosidic bond nature for N-glycan structures on microarrays.

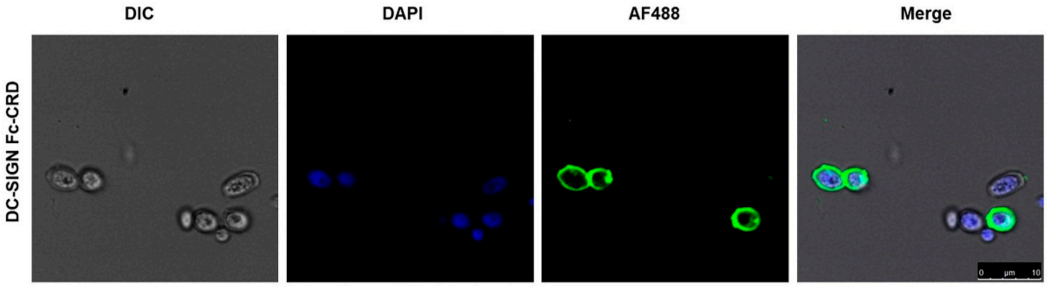


**Figure S2.** Binding of DC-SIGN Fc-CRD to heat-killed *Candida albicans* using confocal microscopy. *C. albicans* cell wall was visualized by differential interference contrast (DIC). *C. albicans* DNA was stained using DAPI (in blue), while the DC-SIGN Fc-CRD construct was detected using the AF488-conjugated secondary antibody (in green). Three random pictures were taken per independent experiment ($n$ = 3). Scale bar indicates 10 µm.

**Table 1.** Glycosidic bond nature of additional glycan structures included on microarrays.

| | | |
|---|---|---|
| GL46 | GlcNAcβ1-2(GlcNAcβ1-4)Manα-sp | |
| GL81 | GlcNAcβ1-4GlcNAcβ-sp | |
| GL89 | Neu5Acα2-3Galβ1-4(Fucα1-3)GlcNAcβ-sp | SialylLeX |
| GL91 | Galβ1-4(Fucα1-3)GlcNAcβ-sp | LeX |
| GL93 | GalNAcβ1-4GlcNAcβ-sp | LDN |
| GL94 | GalNAcβ1-4(Fucα1-3)GlcNAcβ-sp | |
| GL95 | GlcNAcβ-sp | |
| GL96 | GlcNAcβ1-4GlcNAcβ1-4GlcNAcβ-sp | |
| GL97 | GalNAcβ1-4Galβ1-4GlcNAcβ-sp | GG3 |
| GL98 | Galβ1-4Glcβsp | lactose |
| GL99 | Manα1-2Manα1-2Manα-sp | |
| GL100 | Xylβ-sp | |
| GL101 | Glcβ-sp | |
| GL102 | Galβ-sp | |
| GL103 | Fucα-sp | |
| GL104 | Gala1-3GalNAca-sp | |
| GL105 | Galα1-2Galβ-sp | |
| GL106 | Galα1-3Galβ-sp | |
| GL107 | Fucα1-2Galβ-sp | |
| GL108 | Fucα1-3GlcNAcβ-sp | |
| GL109 | Fucα1-4GlcNAcβ-sp | |
| GL110 | Galα1-3Galβ1-4Glcβ-sp4 | |
| GL111 | Galα1-3Galβ1-4GlcNAcβ-sp | |
| GL112 | Fucα1-2Galβ1-3GlcNAcβ-sp | $Le^d$ (H type 1) |
| GL113 | Fucα1-2Galβ1-3GalNAcα-sp | H (type 3) |
| GL114 | Fucα1-2Galβ1-4Glcβ-sp | H (type 6) |
| GL115 | Galβ1-3(Fucα1-4)GlcNAcβ-sp | $Le^a$ |
| GL116 | GalNAcα1-3(Fucα1-2)Galβ-sp | $A_{tri}$ |
| GL117 | Galα1-3(Fucα1-2)Galβ-sp | $B_{tri}$ |
| GL118 | Neu5Acα2-3Galβ1-4Glcβ-sp | 3′SL |
| GL119 | Neu5Acα2-3Galβ1-4GlcNAcβ-sp | 3′SLN |
| GL120 | Neu5Acα2-6Galβ1-4GlcNAcβ-sp | 6′SL |
| GL121 | Fucα1-2Galβ1-3(Fucα1-4)GlcNAcβ-sp | $Le^b$ |
| GL122 | Fucα1-2Galβ1-3(Fucα1-3)GlcNAcβ-sp | $Le^y$ |
| GL123 | Neu5Acα2-3Galβ1-3(Fucα1-4)GlcNAcβ-sp | $SialLe^a$ |
| GL124 | GlcNAcβ1-4(Fucα1-6)GlcNAcβ-sp | $FucGlcNAc_2$ |
